# Prolonged Gestation

**Published:** 1855-01

**Authors:** J. J. Chisolm

**Affiliations:** Charleston, S. C.


*Prolonged Gestation.* Reported by J. J. Chisolm, M. D., of Charleston, S. C.—Towards the close of July, 1843, I was requested to see a healthy, robust servant girl, aged eighteen, of good constitution, whose abdomen was rapidly enlarging, unaccompanied by pain or even uneasiness, caused from sudden cessation of her menses, five months previously. She considered herself dropsical, which alarmed her owner. Suspecting pregnancy, no treatment was instituted except mild laxatives to obviate costiveness, from which she was suffering.

Leaving the city for the summer months, I heard no more of the case, till my return, when I was requested to attend the girl in her delivery, she having confessed her condition when it could no longer be concealed. Towards the end of November, she having, as she supposed, arrived at full term, was attacked with cramps in the abdomen and pain in her back, which lasted an entire day, when they disappeared. In December, the pains and cramps returned with greater severity—so much so, that the patient was crying all the day of the pain. These, also, disappeared, and an interval of a fortnight elapsed, when, in the early part of January, a return of the pains brought on a tedious labor. The enormous size of the child induced me to weigh it, on the second day after birth, when, to my surprise, it weighed fourteen pounds—duplicates of its clothing weighed eight ounces, leaving thirteen and a half pounds as the net weight of the infant. Had it been weighed immediately after birth, it would have been heavier, as it had several copious evacuations and had taken no nourishment.

The case is interesting, from the circumstance of gestation having been prolonged nearly two months over the time, dating from the first appearance of cramps and pain in her back, in November. This is also corroborated by her previous report of suspension of menses, in March, which she attributed to taking cold, and still strengthened by the large size of the child—the parent being rather below the medium stature.—*Charleston Medical Journal, Nov.* 1854.

